# Current News and Opinion

**Published:** 1887-09

**Authors:** 


### Current News and Opinion.

### IS DENTISTRY A SPECIALTY IN MEDICINE?

The relations of dentists to doctors have been the subject of much discussion. Dentistry has grown up from an unsavory beginning; so has surgery. No dentist was ever lower than the barber surgeons of England. The same might be said of most specialties. Hence, dentistry should not be denied recognition in the medical profession because of its lowly origin. The question arises, has it so developed as to merit a place by the side of other specialties? It is well known that for several years some medical schools have undertaken to give the degree of M. D. to such dental students as added to their special dental training a certain culture in the medical sciences. Apparently, with each year the sentiment has grown in the profession and among the better dentists that they were practically one.

Hence, at the late meeting of the American Medical Association, it was voted to recognize such dentists as graduate at dental schools requiring preliminary requirements of a fair literary education, and which compel a three years' training in a first-class college. These requirements are higher than those of most medical colleges, those admitting all who apply, and graduating them after attendance upon a portion of two courses of lectures. Hence, such dental schools as comply with the prescribed requirements will be better trained than the average of the average medical college. This is good for the dentists, but bad for the doctors. It is not too high for good dentists, but it is far too low for good doctors. The INDEPENDENT PRACTITIONER, speaking for progressive dentists, says that the proposition of the American Medical Association is fair. But it says that only a portion of the dental schools comply with it. (Here follows a quotation of a part of the editorial in this Journal for July: "The late action of the American Medical Association.")

It is worthy of note that all here said respecting the dental colleges and dental students applies with equal force to the medical schools and medical students. Hence, dentists should not be denied recognition by the medical profession for possessing the same defects and vices which prevail among doctors.

As a matter of fact, the time is rapidly approaching when the dentists will be quite as much doctors in all regards as are most specialists. There is, under such circumstances, no reason why all should not be called doctors.

More knowledge of dentistry by physicians, and more knowledge of medicine by dentists, would result in mutual benefits.—*American Lancet.*

---

### NEW ENGLAND DENTAL SOCIETY.

The New England Dental Society will hold its 25th annual meeting in Boston, on the 5th, 6th and 7th of October. This is the largest and oldest dental society in New England, and next to the largest in the country. It is intended to make the meeting a celebration of our Silver Wedding. Many eminent men in our profession throughout the country have promised to attend and make the occasion successful. A complete programme of the meeting will be sent to all signifying their intention of attending, at a later date.

A. M. DUDLEY, Secretary.

### PENNSYLVANIA STATE DENTAL SOCIETY.

The Pennsylvania State Dental Society convened at Glenn Summit, July 26 to 28, 1887, inclusive, profitably, no doubt, to all in attendance.

The following officers were elected for the ensuing year :

*President*—W. F. Fundenberg.

*First Vice-President*—W. E. Van Arsdel.

*Second Vice-President*—Louis Jack.

*Recording Secretary*—W. B. Miller.

*Assistant Secretary*—J. R. C. Ward.

*Treasurer*—L. Ashley Faught.

*Chairman Ex. Com.*—S. H. Guilford.

The next annual meeting will be held at Philadelphia, Pa., first Tuesday in June, 1888. R. K. Filbert, Cor. Sec.

---

### CONNECTICUT VALLEY DENTAL SOCIETY.

The annual meeting of the Connecticut Valley Dental Society will be held at "Hotel Warwick," Springfield, Mass., Thursday and Friday, Oct. 28 and 29. A cordial invitation is extended to the profession.

Geo. A. Maxfield, D. D. S., Secretary.

---

The New York Medical Society has been trying for three years to have put on the statute books a measure requiring the licensing and registering of physicians. All the schools of medicine finally united this year on the bill, which has just become a law. It restricts the list of authorized practicing physicians to those already regularly licensed and over 21 years of age. Hereafter, those who shall be admitted to practice will be confined, first, to those who shall have been graduated from an incorporated medical school or college with the degree of Doctor of Medicine; second, to those who shall have received this degree from the Regents of the University of the State of New York; third, to graduates of incorporated medical institutions in other States and foreign countries, which shall have been approved by the institutions in this State. The county clerk of each county shall keep a registry book, in which every physician must register according to a prescribed formula. No person convicted of committing a felony shall be allowed to practice. The penalty for violating the law is $50 fine for the first offense and $100 fine or 100 days' imprisonment, or both, for each succeeding offense. The county medical societies are authorized to prosecute any offenders.

---

Dr. Flavius Searle, of Springfield, Mass., is about to enter upon his jubilee year, having been in practice a half century. What a retrospect is his—what wondrous changes has he witnessed! And now after fifty years in dentistry, his interest in professional advancement is as intense, his desire for knowledge as keen, as when he first set out upon his exceedingly honorable career. How many men for fifty years can keep pace with the progress of the world, especially in a profession that is making such advances as is dentistry ?

A CASE HAVING ARISEN in which a legally qualified practitioner of medicine was prosecuted by the New York State Dental Society for practicing dentistry illegally, Mr. Justice Gorman decided that the medical qualification carried with it the right to practice dentistry. It was urged in behalf of the prosecution that the defendant was a graduate of the Eclectic Medical College, and that he had been convicted of malpractice; but it was shown that, whatever the college might be, it was legally empowered to grant the medical degree, carrying with it the license to practice, and that, as regards the conviction for malpractice, the defendant said that he had been pardoned by the Governor. The judge promised to dismiss the complaint if the defendant brought proof of his having been pardoned —*New York Medical Journal.*

It should be understood that the New York State dental law provides that a diploma from a reputable college, *recognized as such by the Dental Society of the State of New York*, shall be sufficient authority under the law for dental practice, no matter whether it be a dental or medical degree.—*Editor I. P.*

---

A. ZUCKERMANN (*Centralbl. f. Bacteriologie u. Parasitenkunde*, No. 17,) relates his experiments upon suppuration, which have led him to these conclusions: That no chemical, mechanical, or thermic influences can excite suppuration in tissues wholly free from microbes; and in cases where these causes apparently act, it is probably through some pyogenic microbe. Substances chemically pure may be mycotically impure; thus some disinfectants are not always free from microbes. The varieties of microbe known to cause suppuration are *Staphylococcus Pyogenes Aureus*, *Albus*, and *Citreus; Streptococcus Pyogenes;* and in fœtid abscesses, *Bacillus Pyogenes Fœtidus.* Inoculations with Staphylococcus and Streptococcus produce fatal results if injected in large amount into animals. or lead to suppuration if death do not occur. The pyogenic microbes must have a very general distribution in nature; they may enter the body through the air-passages, the intestinal canal, and especially the skin, and by means of small wounds, or the orifices of the cutaneous glands. Staphylococcus is more frequent than the Streptococcus pyogenes.

---

FEW PERSONS have an adequate idea of the immense extent of some of the Western States and Territories. Take Texas for instance. A straight line drawn across the State at its greatest diameter is nearly as long as from New Orleans to Chicago. Place the entire population of the United States (fifty millions) in Texas, and the State would not then be as densely populated as Germany. If the vast agricultural resources of the State were fully developed, it could easily support all that great population. Montana, Idaho, Dakota and New Mexico are almost as large. Of the twenty-two States and Territories west of the Mississippi, only three are as small as all New England. These facts have no direct relation to dentistry, but they suggest possibilities to the young men who are about to crowd into the cities to practice dentistry. Perhaps some of the "old settlers," who are alarmed about the encroachment of dental colleges, could find more elbow room out west.—*Cin. Med. and Dental Journal.*

THE LUELLA MABBITT disappearance case has caused much excitement in Indiana. At the meeting of the Indiana State Dental Society, the skull of a person which, it is claimed, is that of the missing girl, for whose murder her lover is being tried, was produced, and without any knowledge of its history was submitted to the critical examination of the dentists, who were asked to fill out and sign blank certificates, giving their opinion as to the probable age of the person. The average of thirty-one opinions was forty-eight and one-half years. Seventeen pronounced it a male, one a female, while the others did not express an opinion.

*The Review* says that since that time the coroner and three doctors have certified that the body was that of a female, and sufficient of the anatomy has been produced in court to positively establish that they are correct. Even dentists may be mistaken, and they should exercise great care about expressing expert opinions in such grave cases.

---

DR. M. VOGEL, writing upon the subject of cleansing the hands, says that he has noticed that coppersmiths, tinsmiths, etc., whose hands become covered with a dirt from working in oxides and acids that cannot be removed by ordinary means, first rub their hands with warm oil, and when this has thoroughly penetrated, rub them with powdered borax. Subsequent washing with soap and water makes the hands perfectly clean. He advises those who have to use carbolic acid to go through the process above described first, and claims that in this way, (1) disinfection is made more thorough; (2) the hands are made purer than it is possible to make them with soap alone ; (3) the hands remain soft and free from troublesome, rough epidermic scales, and the odor of carbolic acid is destroyed ; (4) the uncomfortable anæsthesia after washing with carbolic acid is avoided.—*Allgemiene Med. Central-Zeitung.*

---

SOME OF THE RECEPTIONS and entertainments to be given in connection with the International Medical Congress meeting in Washington are as follows: Monday evening, Conversazione at U. S. Pension Hall, 8 to 11.

Tuesday evening, visit to the Corcoran Art Gallery.

Wednesday evening, reception by the citizens of Washington, from 8 to 12.

Thursday evening, General Reception and Buffet Banquet at United States Pension Hall, from 8 to 11.

Saturday afternoon, visit to Mount Vernon.

An excursion to Niagara Falls will also be tendered the foreign members of the Congress, with their ladies. The exact time of starting is yet to be announced.

---

THE FIRST ANNOUNCEMENT of the Dental Department of Columbian University, at Washington, has been issued. The professors, aside from the occupants of medical chairs, are: Dental Prosthetics—J. Hall Lewis, D. D. S.; Operative Dentistry—H. C. Thompson, D. D. S.; Demonstrators—H. B. Noble, Jr., D. D. S.; Mark F. Finley, D. D. S.

Attendance upon two courses of five months each will be essential for graduation. The term will begin October 1st prox.

IN THIS NUMBER will be found the advertisement of Dr. D. B. Freeman's Double Loop Spring Clamp, and his Disk Cutters. We are always glad to present to the profession any new device which promises to be useful, and therefore we call attention to these articles. The Spring Clamp we have been using for some weeks, and have found that in filling buccal cavities in the anterior teeth it is an essential. From the very first operation we derived benefit sufficient to pay for a set of the clamps. The device is so simple and so obviously useful that it is but necessary to call attention to it.

For a number of years we have been using a disk-cutter not unlike that of Dr. Freeman, and with it the office girl has constantly kept the operating case supplied, without trouble and almost without expense. But Dr. Stevens' Cutter was not placed regularly on the market, and few knew where to obtain them. A good disk cutter is something more than a convenience, and those made from Dr. Freeman's patterns are excellent.

---

THE AMERICAN DENTAL ASSOCIATION at its late meeting elected the following named gentlemen to serve as officers for the ensuing year.

*President*—Frank Abbott, New York.

*First Vice-President*—C. R. Butler, Cleveland.

*Second Vice-President*—T. S. Waters, Baltimore.

*Recording Secretary*—Geo. H. Cushing, Chicago.

*Corresponding Secretary*—Fred A. Levy, Orange, N. J.

*Treasurer*—Geo. W. Keeley, Oxford, Ohio.

*Members of Executive Committee*—L. D. Shepard, Boston; A. W. Harlan, Chicago; A. O. Hunt, Iowa City.

---

ARCHIVES OF PEDIATRICS relates a case in which an infant of two months of age died after having been for some time subject to terrible abdominal pains. An autopsy revealed that through a small opening in the diaphragm all the large intestines, as far as the cæcum, had passed into the left side of the thorax, filling it full. The left lung had never been expanded, but lay flattened against the spine. The heart lay underneath the border of the right lung, and the spleen was depressed out of position. The extraordinary severity of the abdominal pain was due to the efforts of the bowels to force their contents through the construction produced by the narrow opening in the diaphragm.

---

ONE OF THE GREATEST living authorities upon buccal bacteriology, Dr. Miller, finds that by using the following mixture he can completely sterilize the mouth, cavities in carious teeth, etc. Thymol, 4 grains; benzoie acid, 45 grains; tincture eucalyptus, 3½ fluid drachms; water, 25 fluid ounces. The mouth is to be well rinsed with the mixture, especially just before going to bed, since most of the damage by fermentative and putrefactive processes in the mouth is done at night, during sleep.—*Brit. Journal Dental Science.*

---

TO PREVENT the sticking together of postage stamps that are carried in the pocket or memorandum book, rub the sticky side over the hair. The oil of the hair coats the mucilage and prevents it from sticking.

Judge C. C. Fuller, of Mecosta County, in the case of State of Michigan vs. Vanimmans, decided, when a physician refused to testify on the ground that the evidence would be expert testimony: "After many years' study and observation I decide that a physician's knowledge is his stock in trade, his capital, and we have no more right to take it without extra compensation than we have to take provisions from a grocery, without pay, to feed the jury. The court rules that the witness is not compelled to testify."

---

In Scribner's Magazine for September will appear two essays which will attract general attention and be especially interesting to professors, scholars and students of literature. One is a very carefully elaborated paper on "The Development of the American University," by Professor George T. Ladd, of Yale, who presents a scheme for primary, secondary, and higher education. The other is an essay by Professor Adams Sherman Hill, of Harvard, on "English in Newspapers and Novels."

---

Dr. Geo. H. Rohé, is about to assume the editorialship of a new quarterly journal, devoted to the scientific and practical consideration of questions in the domain of medical and sanitary climatology. It will be called *The Climatologist*, and will be issued from Baltimore. Dr. Rohé has an established reputation as a writer upon preventive medicine, and will bring to the discharge of his duties a thorough knowledge of the subjects to which the new journal will be devoted.

---

Horsford's acid phosphate is a remedy of which all have heard. During the heated term of the past two months we have formed a personal acquaintance with it, and have no fear of saying too much in its favor. When made into a kind of lemonade, it furnishes a refreshing, cooling drink, that is exceedingly grateful. But it is as a remedy for certain gastric disturbances that we have found it most useful, and to its merits we can bear personal testimony.

---

The Saguenay Pilgrims from the Montreal meeting of the Connecticut Valley Dental Society, while on their trip, bought and presented to Dr. Lovejoy a beautiful ice-pitcher, suitably inscribed, as a mark of appreciation of his indefatigable labors in behalf of the meeting. Drs. Andres, Bazin, Beers, Trestler and many others, received no ice-pitchers, simply because there were not enough to go round.

---

There are few, if any cities of its size, that can boast so many medical journals of influence as Detroit. *The Medical Age*, *The American Lancet* and *The Therapeutic Gazette* are among the foremost of American medical publications, and each boasts of a large and constantly increasing subscription list. Besides these three, *The Index Medicus* and *The Druggists' Bulletin* are published by Geo. S. Davis.

---

Let it not be forgotten that the dentists of Western New York will have a reunion in Buffalo, October 25th. The Sixth, Seventh and Eighth District Societies will hold a union meeting, and the Executive Committee is planning things unutterable.

Wm. Wood & Co. make a very courteous offer that is open to any medical journal in the world. They will have a stenographic report made of all the proceedings of the International Medical Congress, and will send a copy to any journal that shall make application for it. This is not only an evidence of enterprise, but it is an instance of journalistic courtesy that is seldon paralleled.

---

Through a curious mental confusion, the brothers W. A. and J. H. Spaulding have exchanged identities in our mind, and some annoying blunders have been the consequence. It was to Dr. W. A. Spaulding, of Minneapolis, that the contribution to the Waite testimonial fund should have been credited Dr. J. H. Spaulding is practicing in Paris, France.

---

Dr. L. P. Haskell, of Chicago, lately met with an accident which cost him the sight of one of his eyes His many friends will be glad to know that the vision of the other eye is unaffected, but for a man who has passed the meridian of life to become habituated to a new method of vision is a serious undertaking.

---

In Germany almost the only anæsthetic used is chloroform. Billroth uses a mixture of chloroform and ether. The German surgeons regard all anæsthetics as dangerous, chloroform no more so than others and they consequently choose it on account of its practical advantages.

---

A recent estimate shows that about one-fourth the population of New York, Boston, and London receives free treatment at the medical clinics and hospitals; in Philadelphia one-fifth, and in Liverpool one-half the population.

---

Dr. Chisholm, according to a medical exchange, thinks that chloroform was made for the skilled, and ether for the blunderers to use. Ether is the dull knife for the boy, while chloroform is the sharp razor for the man.

---

The chief of police of Chicago has issued an order giving to the carriages of physicians precedence at the bridges, classing them with mail and patrol wagons, ambulances and fire apparatus.

---

The Association of American Medical Editors will tender a banquet to the foreign editors and other distinguished guests at Washington, Wednesday evening, Sept. 5, 1887.

---

If any first-class dentist desires to secure the leading practice in Constantinople, he should correspond with Dr. P. L. Foote, of Poughkeepsie, N. Y.

---

Fairmont, Ind., has a natural gas well, lately opened, that flows nearly 12,000,000 cubic feet per day.

---

The United States has twice as many doctors in proportion to its population as has England.

---

A doctor may understand only one language, and yet know several tongues.

---

The practitioner who is jealous of a rival acknowledges his own inferiority.

